# Meta-analysis of the clinical and immunopathological characteristics and treatment outcomes in epidermolysis bullosa acquisita patients

**DOI:** 10.1186/s13023-018-0896-1

**Published:** 2018-09-04

**Authors:** Hiroaki Iwata, Artem Vorobyev, Hiroshi Koga, Andreas Recke, Detlef Zillikens, Catherine Prost-Squarcioni, Norito Ishii, Takashi Hashimoto, Ralf J. Ludwig

**Affiliations:** 1 Department of Dermatology, University of Lübeck, Ratzeburger Allee 160, D-23538 Lübeck, Germany; 2 Department of Dermatology, Kurume University School of Medicine, and Kurume University Institute of Cutaneous Cell Biology, Kurume, Fukuoka, Japan; 3 Referral center for auto-immune bullous diseases, Department of Dermatology, APHP, Avicenne Hospital, Bobigny, France; 4 Department of Dermatology, Faculty of Medicine, Osaka City University, Osaka, Japan; 5 Present address: Department of Dermatology, Hokkaido University Graduate School of Medicine, Sapporo, Japan; 6 Lübeck Institute of Experimental Dermatology, University of Lübeck, Lübeck, Germany

**Keywords:** Epidermolysis bullosa acquisita, Treatment, Meta-analysis, Diagnosis, IVIG, Rituximab

## Abstract

**Background:**

Epidermolysis bullosa acquisita (EBA) is an orphan autoimmune disease. Several clinical phenotypes have been described, but subepidermal blistering is characteristic of all variants. Limited data on clinical and immunopathological characteristics and treatment outcomes in EBA are available. To fill this gap, we collected this information from EBA cases, meeting current diagnostic criteria, published between 1971 and 2016.

**Results:**

We identified 1159 EBA cases. This number must be, however, interpreted with caution, as it is not possible to check for multiple reporting. The analysis of all cases indicated that EBA affects all age groups (median: 50 years, range: 1 to 94 years) at an equal gender distribution. Non-mechanobullous (non-MB) forms of EBA were observed in 55% of patients, whereas the mechanobullous variant (MB-EBA) or a combination of both variants was described in 38 or 7% of patients, respectively. Type VII collagen (COL7)-specific autoantibodies were primarily of the IgG isotype, but anti-COL7 IgA, IgM and IgE were also documented. Comparison of the 2 clinical EBA types showed a higher frequency of IgA deposits in non-MB EBA as opposed to MB EBA. Mucous membrane involvement was observed in 23% of patients, and 4.4% of cases were associated with other chronic inflammatory diseases. Of note, IgA deposits were more frequently observed in cases with mucous membrane involvement. Our analysis indicated that EBA is difficult to treat and that the choice of treatment varies widely. Chi square was applied to identify medications associated with complete remission (CR). Considering all EBA cases, intravenous immunoglobulin (IVIG, *p* = 0.0047) and rituximab (*p* = 0.0114) were associated with CR. Subgroup analysis demonstrated that no treatment was associated with CR for non-MB EBA, while IVIG (*p* = 0.003) was associated with CR in MB EBA.

**Conclusions:**

Within the limitations of the study**,** we here document the clinical and immunopathological characteristics and treatment outcomes in a large cohort of EBA patients. The observed associations of single drugs with treatment outcome may serve as a guide to develop clinical trials.

**Electronic supplementary material:**

The online version of this article (10.1186/s13023-018-0896-1) contains supplementary material, which is available to authorized users.

## Background

Epidermolysis bullosa acquisita (EBA) was first used as a descriptive diagnostic term for the adult onset of a disease resembling epidermolysis bullosa dystrophica at the beginning of the twentieth century [1]. In 1971, Roenigk et al. established the first diagnostic criteria for EBA. An EBA diagnosis depends on the following criteria: (i) clinical lesions resembling epidermolysis bullosa dystrophica; (ii) adult onset of disease; (iii) a negative family history of epidermolysis bullosa dystrophica; and (iv) exclusion of other bullous diseases [2]. In 1973, Kushniruk first noted the deposition of IgG and C3 along the dermal-epidermal junction in EBA patients [3]. These immune deposits were located beneath the lamina densa in the anchoring fibril zone as determined by immunoelectron microscopy (IEM); clearly in a different localization than immune deposits observed in patients with bullous pemphigoid [4, 5]. Subsequently, a putative 290 kD autoantigen located at the skin basement-membrane was identified [6] and later recognized as type VII collagen (COL7), the major component of anchoring fibrils at the dermal-epidermal junction [7]. The pathogenicity of autoantibodies targeting COL7 has been independently demonstrated both in vitro, ex vivo and in vivo [8–11]. Hence, EBA is classified as an organ-specific autoimmune disease. Based on this understanding, the detection of tissue-bound antibodies at the basement membrane zone in specimens from peri-lesional skin or mucous membrane biopsies and autoantibodies specific to COL7 is the current standard for EBA diagnosis [12–14]. Previously direct IEM was the gold standard for a definite EBA diagnosis. It is still an alternative in seronegative EBA. Based on the specific COL7 expression pattern, EBA can also be diagnosed via detection of a u-serrated pattern by direct IF microscopy [15] or Fluorescent Overlay Antigen Mapping (FOAM) [16].

The clinical presentation of EBA is diverse. In the mechano-bullous (MB, non-inflammatory, classical) disease variant, patients suffer from skin fragility, tense blisters, scarring and milia formation primarily localized to trauma-prone sites and the extensor skin surface. In these patients, nail dystrophy, post-inflammatory hyper- and hypopigmentation are also frequently observed. In mild cases, the clinical presentation is similar to porphyria cutanea tarda, whereas severe cases are comparable to hereditary recessive dystrophic epidermolysis bullosa. EBA can also resemble other autoimmune bullous dermatoses (AIBD), such as bullous pemphigoid (BP), linear IgA disease (LAD), mucous membrane pemphigoid (MMP) or Brunsting–Perry pemphigoid. In these patients, widespread vesiculobullous eruptions are observed, typically involving the trunk, central body, extremities and skin folds. The patients typically suffer from pruritus. These variants are categorized as non-MB EBA [14, 17–21]. An individual patient may present with either one of these variants alone or in combination. In addition, a patient’s clinical presentation may change from one variant to the other during the disease course [8]. However, data on the prevalence of the different phenotypes of EBA are not available.

Given that COL7 is expressed in the gastro-intestinal tract, the involvement of the oral cavity and other mucosal sites has been frequently reported – and thus EBA must be considered a mucocutaneous disease [14, 20, 22–24]. In addition, other mucous membrane involvement, e.g. ocular and genital, have been repetitively noted in EBA patients, and extracutaneous involvement may occur more often than currently recognized given that a detailed evaluation of mucosal involvement by a multidisciplinary team of medical care providers indicated extensive mucosal involvement [25, 26]. Again, a comprehensive overview on mucosal involvement and affected organs is not available.

In addition to concomitant mucosal involvement, EBA has also been reported to be associated with cancer as well as inflammatory, infectious, cardiovascular, metabolic and neurological diseases [21, 27–33]. However, most of these findings are case reports, and no clear pathogenetic interaction between EBA and these diseases has been established. By contrast, accumulating evidence suggests an association between EBA and inflammatory bowel diseases (IBDs), such as ulcerative colitis (UC) and Crohn’s disease (CD). IBD is reported to be present in approximately 30% of EBA patients. CD is associated with EBA in at least 25 cases [23, 34, 35] and four EBA cases have been reported to be associated with UC [35]. In EBA patients with CD, circulating COL7 antibodies have been noted in frequencies ranging from 6 to 60% [23, 36, 37]. However, these findings must be interpreted with caution as many of these observations were made before the modern diagnostic criteria for EBA were established [38–40]. Further evidence of a pathogenic link between IBD and EBA was obtained from EBA mouse models. In both antibody transfer-induced and immunization-induced EBA, blister formation was observed in the esophagus, stomach, small intestine, and colon in addition to the skin [24]. The prevalence of blister formation in these mouse models parallels COL7 expression, which decreases from proximal to distal regions of the gastrointestinal tract. This anti-COL7-induced gastrointestinal tissue injury is functionally relevant as weight loss or failure to gain weight appropriately gain weight was noted in diseased mice [24].

Despite several in depth reviews on EBA [41–43], detailed insights into the epidemiological, clinical and immunological characteristics of EBA patients on a larger scale are not available. However, this information would be valuable for coordinating standardized diagnostic and therapeutic interventions as well as planning future clinical trials. Therefore, we collected these data from all EBA cases published from 1971 to 2016 that fulfilled the current diagnostic criteria.

## Methods

### Search strategy and selection

We searched PubMed using the term “(epidermolysis bullosa acquisita) AND (“1971”[Date - Publication]: “2016”[Date - Publication]).” From this search, case reports, case report series and experimental studies with patient material were considered for further evaluation. Patients from full text articles were considered if they displayed linear Ig deposits via direct IF microscopy of a skin biopsy *or* immunoelectron microscopy findings of EBA and fulfilled any of the following criteria: (i) detection of anti-COL7 antibodies (any method) or a 290 kD band via western blotting of dermal extracts ([44]); (ii) detection of a u-serrated pattern in direct IF microscopy [15]; (iii) FOAM; or (iv) split mapping techniques [4, 16, 45]. If documented, the age, gender and ethnic background of each patient was recorded. In addition, information regarding clinical EBA phenotype (MB, non-MB or both), mucous membrane involvement (any, ocular, oral, esophagus, laryngeal, pharyngeal, anal, or genital), associated diseases (any), circulating and tissue-bound anti-COL7 Ig isoforms, the observed serration pattern via direct IF microscopy and applied treatments, including outcomes, were documented from the publications.

### Statistical analysis

Descriptive statistical analysis was performed using Excel. The determination of traits associated with clinical EBA variants or mucous membrane involvement was performed using Chi square test. Treatment outcomes were classified as complete remission (on or off therapy), partial remission (on or off therapy) and no response. Chi square test was then used to identify those treatments associated with complete clinical remission. This analysis considered single compounds. Hence, this analysis does not evaluate effects of combination therapy. Furthermore lastly, we did not differentiate between complete remission on or off treatment.

## Results

### Selection of EBA patients

**Fig. 1:**
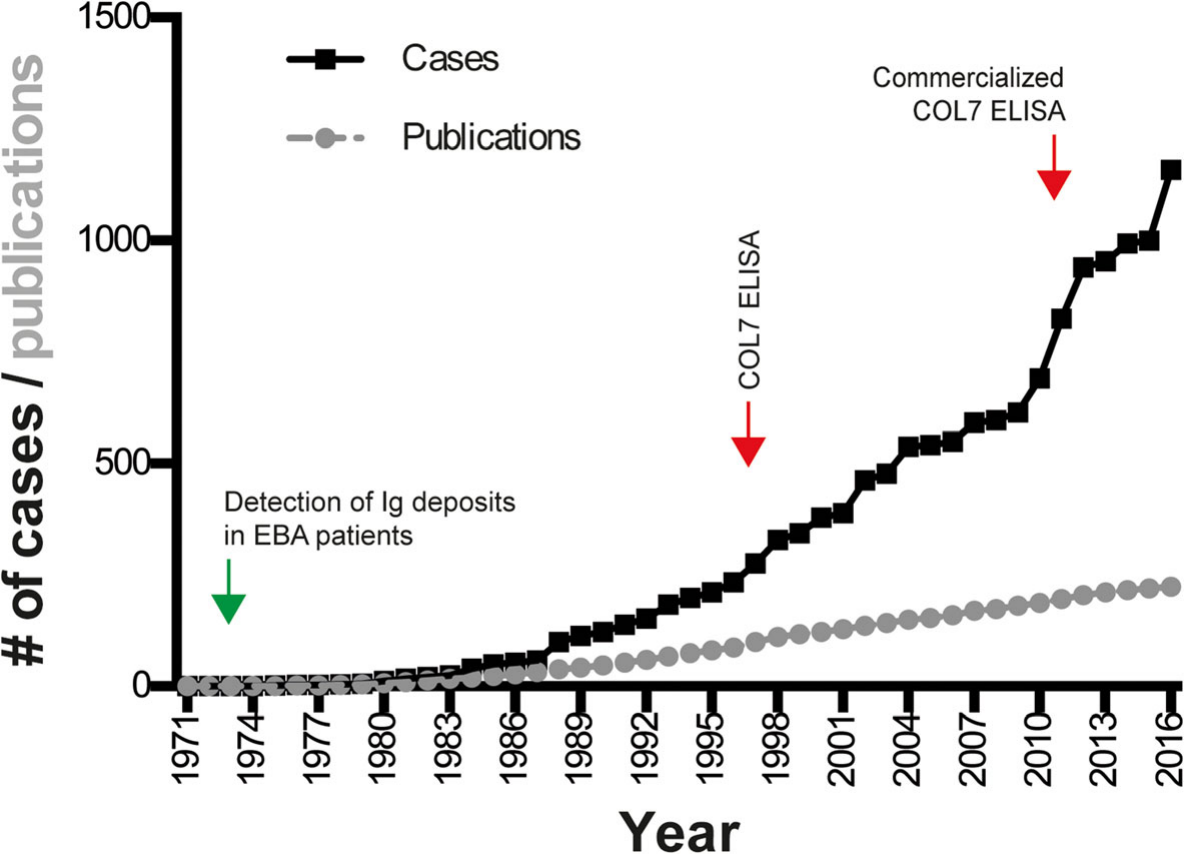
Reported EBA cases and number of publications reporting EBA patients from 1971 to 2016. PubMed was searched using the term “(epidermolysis bullosa acquisita) AND (“1971”[Date - Publication]: “2016”[Date - Publication])”. EBA patients fulfilling the current diagnostic criteria were selected from the retrieved records. A total of 1159 EBA cases (data sets) were identified. Over the years, the number of reported cases ranged from 2 to 5 per year with the exception of 1996–99 and 2011–12, when 11–62 patients were reported per year. The graph displays the cumulative number of EBA patients reported between 1971 and 2016. The number of publications on EBA patients remained relatively constant during the time frame assessed; during this time, 2–6 manuscripts per year were typically published. If publications with a focus on immunological studies, i.e. ELISA development or HLA-associations, are excluded from this analysis, a total of 519 data sets in 194 publications remain. The green arrow indicates the time point when IG deposits were first noticed in EBA patients [3], while the red arrow corresponds to the description of the first [47] and the first commercialized [72] ELISA system detecting autoantibodies directed against COL7

Our search yielded 761 publications. Of these, 410 publications were identified that potentially contained information on EBA patients as determined by reading titles and abstracts. After reading the full text articles, 1159 EBA cases from 224 publications were included in our analysis (Additional file 1: Table S1). Based on the data provided, it is not possible to rule out that some patients were reported in several publications. The remaining cases were excluded if the study did not sufficient provide information on the specific patient(s) or the reported cases failed to meet the current EBA diagnostic criteria. The number of publications as well as the number of reported EBA cases remained relatively constant from 1979 to 2014. An increased number of EBA patients was reported in 1996–1998 [46–48], 2011–2012 [17, 21, 49] and 2016 [50, 51], which was due to the appearance of new diagnostic tools and thus the publication of large EBA patient collections during these time periods (Fig. 1).

### Age, gender and clinical characteristics of EBA patients

All age groups were affected in this retrospective cohort of EBA patients. The youngest patients at time of diagnosis were one-year-old [52–54], whereas the oldest patient was 94-years-old [55]. The median age of all patients was 50 years (mean: 46.7 ± 22.1 years, Table 1). Of these, 54 (4.6%) patients were children aged 17 years or younger, and 132 (11.3%) patients were aged 65 years or older. Both genders were equally affected by EBA, although slightly (54%) more women suffered from the disease (Table 1). The majority of patients presented with non-MB EBA (55%). Moreover, 38% presented with MB EBA, and 7% displayed characteristics of both EBA variants (Table 1). Furthermore, no differences regarding the clinical EBA phenotypes were noted among different ethnicities (data not shown). Notably, 23% of EBA patients suffered from mucosal involvement; now termed MM EBA [14]. In most cases, the oral mucosa was affected. In EBA patients with mucosal involvement, most patients had a single mucosal site affected, whereas in approximately 30% manifestations in two or more mucosal sites were present. A total of 9.6% of all EBA patients experienced an additional diagnosis. EBA was most commonly associated with other chronic inflammatory diseases. Among these diseases, CD (0.9%), other AIBD (0.6%), thyroiditis (0.4%) and rheumatoid arthritis (RA) (0.5%) occurred most frequently (Table 1). Furthermore, antinuclear antibodies (ANAs) were described in 1.7% of the EBA patients. Yet, most studies did not specify whether ANAs were tested. If the analysis is restricted to reports in which ANA reactivity was specified (i.e., [4, 21, 56]), ANAs were detected in 20 of 80 (20.0%) EBA patients.

**Table 1:** Age, gender and clinical presentation of EBA patients

Age (years)^b^			
- Median	50		
- Range	1–94		
- Mean	46.7		
- Std Dev^a^	22.1		
Gender^c^			
- Female	54%		
- Male	46%		
Clinical phenotype^d^			
- Non-MB	55%		
- MB	38%		
- Both	7%		
Mucosal involvement (any)^e^		Mucosal involvement (specific)^f^	
- Yes	23%	- Ocular	14.1%
- No or not indicated	77%	- Oral	90.8%
		- Esophagus	11.5%
		- Trachea/larynx	8.8%
		- Anal	3.0%
		- Genital	14.1%
Associated diseases^g^		Associated inflammatory diseases^g^	
- Any	9.6%	- CD	0.9%
- Inflammatory	4.4%	- RA	0.5%
- Metabolic	1.3%	- Thyroiditis	0.4%
- Infection	0.9%	- UC	0.6%
- Cancer	1.9%	- Psoriasis	0.4%
- Cardiovascular	0.6%	- DTH	0.1%
- Neurology	0.5%	- Acquired hemophilia	0.1%
- Other	0.2%	- AIBD	0.6%
		- SLE	0.4%
		- Nephritis	0.1%
		- ITP	0.1%

^a^*Abbreviations: Std Dev* standard deviation, *non-MB* non-mechanobullous EBA variant, *MB* mechanobullous EBA variant, *CD* Crohn’s disease, *RA* rheumatoid arthritis, *UC* ulcerative colitis, *DTH* delayed type hypersensitivity, *AIBD* autoimmune bullous dermatoses, *SLE* systemic lupus erythematosus, *ITP* idiopathic thrombocytopenic purpura

^b^Specified in 384 cases

^c^Specified in 561 cases

^d^Specified in 366 cases

^e^Reported in 261 cases

^f^As multiple sites were affected in some patients, the sum of specific mucosal involvement is greater than the reported 23% of patients experiencing any mucosal involvement

^g^Reported in 97 cases. The sum of individual associated diseases may be higher than the indicated total due to multiple associated diseases

### Characterization of COL7 autoantibody responses in EBA patients

In the majority of patients, immunoglobulin (Ig) deposition along the dermal-epidermal junction was observed. IgG deposits were most frequently found, whereas IgA or IgM deposits were detected less frequently, and IgE deposits were rarely reported (Table 2). IgG was the only deposited Ig in 62.1% of cases. IgA, IgM and IgE were exclusively deposited in 2.4, 0.3 and 0.0% of cases, respectively. Complement activation, assayed by linear C3 deposits along the dermal-epidermal junction, was observed in one third of the patients. Age and gender did not display a difference regarding C3 deposition. Circulating anti-COL7 Ig of all subclasses was less frequently detected compared with tissue-bound autoantibodies (Table 2).

**Table 2:** Characterization of the COL7 autoantibody response in EBA patients

	DIF^a^	Circ.	Variant		Age^b^		Gender		MM-EBA	NON-MM EBA
			NON-MB	MB	≤ median	> median	Female	Male		
IgA	8.9%	2.3%	35.7%*	6.9%*	21.1%	17.5%	18.1%	14.7%	16.8%*	7.2%*
IgE	0.3%	0.0%	0.0%	0.5%	1.3%	0.0%	0.3%	0.8%	1.2%*	0.0%*
IgG	78.3%	66.9%	75,2%	82.1%	90.9%	87.5%	88.4%	91.9%	49.9%	48.9%
IgM	5.3%	0.2%	13.3%	10.0%	14.6%	10.4%	12.8%	7.7%	12.1%*	3.7%*
C3	37.8%	N/A	58.4%	42.1%	75.8%	65.4%	53.4%	57.3%	15.2%	22.5%

The numbers in the table correspond to the percentage of the respective Igs detected by direct IF microscopy (DIF) and circulating Ig (Circ.), detected by indirect IF microscopy, ELISA and/or Western blot analysis. Furthermore, the direct IF microscopy findings are also compared between NON-MB-EBA and MB-EBA, i.e., IgA is detected by direct IF microscopy in 24.7% of non-MB EBA patients, whereas IgA tissue deposits are only observed in 9.9% of MB EBA cases. Ig reactivity by direct IF microscopy is also assessed according to patient age (differentiated by the median age of the cohort) and gender as well as mucosal involvement. The lack of standardized diagnostics for EBA and missing details on which tests were performed, is a limitation of the table. Since, based on our experience, direct IF microscopy includes IgA, IgG and C3 in most laboratories, the data for these 3 parameters is most likely very valid

^a^*Abbreviations: DIF* direct immunofluorescent microscopy, *Circ.* circulating immunoglobulin, *Variant* EBA variant, *Mucosal* mucosal involvement

^b^Median age: 50 years

*p < 0.05 (Chi square)

### Immunological differences between MB and non-MB EBA as well as between mucous membrane (MM) and non-MM EBA

We next evaluated whether EBA phenotypes or mucous membrane involvement are associated with certain clinical and/or immunological findings, such as presence of IgA reactivity against COL7, prevalence of circulating IgG or presence of C3 deposits between MB and non-MB EBA. As reported earlier in a smaller sample size [17], we also noted a higher frequency of IgA deposits in non-MB EBA as opposed to MB EBA. Furthermore, IgG, IgM and C3 deposits were more frequent in non-MB EBA (Table 2). Evaluating the presence of IgA deposits in direct IF between MM and non-MM EBA, IgA deposits were reported in 16.8% of MM EBA and in 7.2% of non-MM EBA (Table 2). An increased frequency of IgE and IgM, but not IgG, deposits in MM EBA as compared to non-MM EBA was also noted (Table 2).

### Identification of drugs associated with complete remission

Most reported EBA patients had received multiple treatments owing to the inefficacy of previous treatment(s). Furthermore, a combination of several medications was used in most EBA patients. To obtain insight into potentially effective EBA treatments, we applied Chi square test. This model determined which single treatment, independent of other medications or procedures, was associated with complete remission both on and off EBA treatment. Considering all EBA cases, intravenous immunoglobulins (IVIG) and rituximab were significantly associated with clinical remission independently of clinical EBA phenotype (Table 3). The subgroup analysis of non-MB EBA and MB EBA indicated that the response to treatment is different between these EBA variants: In non-MB EBA no significant associations of complete remission with any given treatment was observed. In MB-EBA, IVIG was associated with complete remission (Table 3, Additional file 2: Table S2). Regarding the dosing and outcomes of IVIG and rituximab treatment, details are provided in Additional file 2: Table S2).

**Table 3:** Association of treatment with complete remission

Treatment	All EBA cases	NON-MB-EBA	MB-EBA	Cases
Corticosteroid	−^a^	–	–	223 | 88 | 30
Dapsone	–	–	–	110 | 43 | 25
Azathioprine	–	–	nd^b^	41 | 11 | 7
Colchicine	–	–	nd	29 | 15 | 7
Cyclosporine	–	nd^c^	nd	30 | 9 | 8
Mycophenolate	–	nd	nd	18 | 2 | 8
IVIG^c^	0.0047	0.003	–	30 | 11 | 13
Methotrexate	–	nd	nd	12 | 7 | 3
Cyclophosphamide	–	nd	nd	10 | 3 | 8
Rituximab	0.0114	nd	nd	16 | 3 | 6

The table indicates which single treatment, independent of other medications or procedures, was associated with complete remission both on and off EBA treatment. The column “Cases” indicates the number of patients reported to be treated with the indicated drug for all EBA cases | non-MB EBA | MB EBA. Addition of the later 2 may be different from all EBA cases, as the type of EBA was not specified for all cases. Chi square test was used to calculate possible statistical significance

^a^Not significant

^b^nd: not done, because less than 10 treated cases

^c^high-dose intravenous immunoglobulin. Only treatments with 10 or more patients were included for statistical analysis. Because of too few reported cases, outcomes for immunoadsorption or immunoapheresis (*n* = 4), daclizumab (*n* = 4), extracorporal photopheresis (*n* = 6) and sulfasalazine (*n* = 1) were not included in this table. The data on the respective treatment outcomes, is, however, listed in the Additional file 1: Table S1

## Discussion

Our meta-analysis documents the clinical and immunopathological characteristics from EBA patients published between 1971 and 2016. The evaluation of treatment outcomes provides insights on the efficacy of current EBA treatments.

From a literature search to establish our meta-analysis cohort, we found several EBA case reports in which the diagnosis could not be validated based on our pre-defined inclusion criteria. When EBA is clinically considered as a potential differential diagnosis, it should only be diagnosed if in addition to linear Ig deposits via direct IF microscopy of a skin biopsy *or* immunoelectron microscopy findings of EBA and fulfill any of the following criteria: (i) detection of anti-COL7 antibodies (any method) or a 290 kD band via western blotting of dermal extracts [44]; (ii) detection of a u-serrated pattern in direct IF microscopy [15]; (iii) FOAM; or (iv) split mapping techniques [4, 16, 45]. When determining the exact diagnosis of EBA, the inclusion of defined criteria for its subtypes is crucial for planning and conducting interventional clinical trials; moreover, an international consensus and standard should be established. Yet, these selection criteria may have led to non-inclusion of “true” EBA cases into the analysis. For example, cases reported with typical clinical features, linear IgG deposits in direct IF microcopy and dermal binding of patient IgG to the dermal side of salt spit skin [57, 58]. Furthermore, differentiation of EBA from LAD with anti-COL7 IgA autoantibodies [59] or “MMP” patients with autoimmunity to COL7 may differ among institutions. Herein, we applied the recently established diagnostic criteria for EBA [14, 59] to differentiate between these diseases.

With the exception of a trend towards the detection of IgA deposits by direct IF microscopy in non-MB EBA but not in MB EBA [17], no laboratory parameter has been reported to be able to distinguish between these EBA variants [8]. In the cohort evaluated in this meta-analysis, IgA deposits, as well as IgG, IgM and C3 deposits, were also more frequently observed in non-MB EBA (Table 2). Furthermore, additional laboratory parameters analyzed in this study could not be used to distinguish between these EBA variants. In addition, IgA deposits were also observed more frequently in MM EBA as opposed to EBA patient without MM involvement. Here, we documented a high prevalence of mucous membrane involvement in EBA, which confirms findings from a previous investigation of four EBA patients [25]. We also believe that inclusion of duplicate cases (i.e. in serological studies) may have “diluted” the prevalence of mucous membrane involvement in our analysis, and that the frequency of this complication is more frequent. Therefore, EBA patients should be monitored for mucous membrane involvement at regular intervals.

Based on the prevalence of autoimmune and chronic inflammatory diseases associated with EBA [60], the observed occurrence of EBA with CD, UC, and other AIBD appears to be higher than expected, whereas other reported EBA-associated diseases seem to occur at rates that are similar to those in the general population. Of note, the here-observed frequency of CD and UC association with EBA is much lower to previous reports, where CD/UC IBD has been reported to be present in approximately 30% of patients with EBA. But some of these observations were made before modern diagnostic criteria for EBA had been established [24]. Hence, the association of EBA with CD and UC seems likely, but needs to be determined prospectively. Interestingly, ANAs were detected in 20.0% of EBA patients, whereas ANA prevalence in healthy controls ranges between 8 and 24% [61–65]. Thus, ANA reactivity seems increased in EBA patients. This observation may indicate that EBA, like pemphigus [66], shares early pathogenic events systemic with systemic lupus erythematous. This notion is strengthened by the clinical disease entity of bullous lupus erythematous [17], an autoantibody-mediated (mostly anti-COL7) subepidermal blistering disease that occurs in patients with systemic erythematosus. Again, as stated above, the methodology used herein most likely underestimates the comorbidity in EBA patients.

Most importantly, our meta-analysis detected significant differences regarding the efficacy of current EBA treatments. First, we document significant variations in EBA treatments, confirming a previous report [67]. Despite the limitations of our analysis, i.e., retrospective nature of the study, inhomogeneity, exclusion of potentially “true” EBA cases (see above) with reported treatment outcomes from the analysis (especially those relating to cyclosporine), and publication bias from the case report primary data, this meta-analysis provides insights into therapeutic efficacy in a large collection of EBA patients. Based on the results from our meta-analysis, which only computed associations of single treatments with the induction of clinical remission, independent of any possible combinatory treatments, IVIG and rituximab seem likely candidates to be used in combination therapies as a treatment for EBA patients. Other associations of treatment efficacy are based on too few cases to draw further conclusions; i.e. 6 cases treated with extracorporeal photopheresis (ECP, Additional file 1: Table S1). This information, as mentioned above, has to be interpreted with caution, but may be useful to guide the planning of clinical trials in EBA patients. To establish the rationale for a controlled clinical trial in EBA patients, the therapeutic efficacy of “established” and emerging EBA treatments should be evaluated in parallel using animal models of the disease [10, 11, 68]. Use of these models has identified several compounds with therapeutic efficacy, including IVIG, as well as potential therapeutic targets [69–71]. The results from coordinated observational studies and therapeutic interventions in animal models will hopefully serve as a basis for the design of a controlled clinical trial in EBA patients. Yet, again, the strict inclusion criteria may have led to non-inclusion of treated EBA patients, which may have had an impact on the analysis.

## Conclusions

In summary, based on the meta-analysis of case reports and case report series, we provide insights into the clinical and immunopathological characteristics and treatment outcomes in all published EBA cases from 1971 to 2016. In addition, this study seeks to improve our understanding of EBA pathogenesis and the limited treatment options. There is a great need to establish an international EBA patient registry, including a collection of prospectively collected biomaterials to foster our further understanding of this disease.

## Additional files

Additional file 1: Raw data. (XLSX 91 kb)

Additional file 2: Raw data. (XLSX 12 kb)
